# Rediscovery of an Endemic Vertebrate from the Remote Islas Revillagigedo in the Eastern Pacific Ocean: The Clarión Nightsnake Lost and Found

**DOI:** 10.1371/journal.pone.0097682

**Published:** 2014-05-16

**Authors:** Daniel G. Mulcahy, Juan E. Martínez-Gómez, Gustavo Aguirre-León, Juan A. Cervantes-Pasqualli, George R. Zug

**Affiliations:** 1 Department of Vertebrate Zoology, National Museum of Natural History, Smithsonian Institution, Washington DC, United States of America; 2 Instituto de Ecología, Asociación Civil, Red de Interacciones Multitróficas, Xalapa, Veracruz, México; Fordham University, United States of America

## Abstract

Vertebrates are currently going extinct at an alarming rate, largely because of habitat loss, global warming, infectious diseases, and human introductions. Island ecosystems are particularly vulnerable to invasive species and other ecological disturbances. Properly documenting historic and current species distributions is critical for quantifying extinction events. Museum specimens, field notes, and other archived materials from historical expeditions are essential for documenting recent changes in biodiversity. The Islas Revillagigedo are a remote group of four islands, 700–1100 km off the western coast of mainland México. The islands are home to many endemic plants and animals recognized at the specific- and subspecific-levels, several of which are currently threatened or have already gone extinct. Here, we recount the initial discovery of an endemic snake *Hypsiglena ochrorhyncha unaocularus* Tanner on Isla Clarión, the later dismissal of its existence, its absence from decades of field surveys, our recent rediscovery, and recognition of it as a distinct species. We collected two novel complete mitochondrial (mt) DNA genomes and up to 2800 base-pairs of mtDNA from several other individuals, aligned these with previously published mt-genome data from samples throughout the range of *Hypsiglena,* and conducted phylogenetic analyses to infer the biogeographic origin and taxonomic status of this population. We found the Isla Clarión population to be most closely related to populations in the Sonora–Sinaloa state border area of mainland México and Isla Santa Catalina, in the Gulf of California. Based on genetics, morphology, and geographic distributions, we also recognize these two other lineages as distinct species. Our study shows the importance of museum specimens, field notes, and careful surveys to accurately document biodiversity and brings these island endemics (Clarión and Santa Catalina nightsnakes) and mainland population near the Sonora–Sinaloa state border to the attention of conservation biologists currently monitoring biodiversity in these fragile subtropical ecosystems.

## Introduction

Current reports of vertebrate extinction events are approaching astonishing levels, with recent estimates of nearly one-fifth of species being listed as Threatened in International Union for Conservation of Nature (IUCN) Red List and some model estimates approaching a nearly 50% extinction rate [1]–[2]. Many of the causes attributed to recent extinction events are global warming, destruction or loss of habitat, infectious diseases, and human introductions of exotic species–reptiles are no exception to this phenomenon [3]–[4]. Island ecosystems are particularly vulnerable to such disturbances and often contain endemic species because of remote access and rare colonization events [4]. However, careful documentation of current species occurrences and distributions are critical for quantifying and validating recent extinction and rediscovery events [2], [5]–[6]. Species distribution records from voucher-based museum collections, archived field notes, and other accounts from natural historians provide invaluable resources for documenting changes in biodiversity [7]–[9].

In 1936, the renowned naturalist William Beebe set sail on the Zaca yacht for a three month expedition from San Diego, California down the Baja California Peninsula to the Cape of San Lucas and out to Isla Clarión in the Revillagigedo Archipelago ∼1100 km from the mainland, collecting marine invertebrates, fishes, birds, and reptiles [10]. Upon his return, specimens were deposited at the American Museum of Natural History (AMNH) in New York; his field notes and travel logs were archived at the New York Zoological Society. Among the many marine invertebrates, fishes, and birds collected, Beebe also collected six reptiles, including a nightsnake (*Hypsiglena*) from Isla Clarión. A decade later, this specimen was described as a distinct subspecies *Hypsiglena ochrorhyncha unaocularus* [11]. However, in 1955, after a brief visit to Isla Clarión and because no other expeditions had encountered this taxon, Brattstrom [12] proposed that the Clarión record was a locality error, and *Hypsiglena* was not considered a faunal component of the island. Subsequent expeditions also failed to encounter this snake, and its potential occurrence was ignored [13]. Thus, the existence of the Clarión Nightsnake was lost to the scientific community, and its presence continued to elude biologists [13]–[14].

Based on Beebe’s specimen, archived field notes, and other accounts of his expedition, we re-evaluated the status of the Clarión Nightsnake with a recent expedition to the island in search of these secretive, nocturnal snakes. We rediscovered this snake on Isla Clarión and collected mitochondrial DNA (mtDNA) sequence data from multiple individuals and a complete mitochondrial genome (mt-genome) from an Isla Clarión *Hypsiglena* and another mt-genome from a closely related population, to compare with previously published data available for the genus [15]–[16]. We evaluate the specific-status of this population and infer its origin of colonization. We emphasize the importance of museum collections and archived materials to properly document biodiversity. We bring the Clarión Nightsnake to the attention of conservation biologists so that efforts can be made to maintain vertebrate diversity in this fragile subtropical island ecosystem.

## Methods

### Ethics Statement

Fieldwork was approved by the Dirección General de Vida Silvestre at the Secretaría de Medio Ambiente y Recursos Naturales (SEMARNAT) and followed the agency’s regulations to avoid unnecessary suffering to animals, required for obtaining the permits [SGPA/DGVS/11040/12 and SGPA/DGVS/04196/13 (SEMARNAT) and SATI/PC/001/13 (SEGOB)]. Specimens were hand captured and voucher specimens were euthanized with 20% benzocaine. Our permits allowed us to collect up to five voucher specimens and the remaining individuals were scored for scalation and color patterns, photographed, tail-tips were taken for genetic analyses (stored in 95% ethanol), and subsequently released at site of capture. Liver or muscle tissue samples were stored in 95% ethanol and specimens were fixed in 10% formalin following standard protocols. Voucher specimens were deposited in the Instituto de Biología, Colección Nacional de Reptiles y Anfibios (CNAR-IBH). Photographic vouchers were deposited in the United States National Museum, Smithsonian Institution (USNM).

### Sampling Methods

We examined the holotype (AMNH-R62756) of *Hypsiglena ochrorhyncha unaocularus* and compared scalation and color pattern with published accounts of the genus. We obtained digital copies of William Beebe’s field notes during the 1936 Zaca Expedition from the Wildlife Conservation Society (formerly the New York Zoological Society), specifically the “*Templeton Crocker Expedition records, Miscellaneous reptiles and amphibia 1936*” and examined other available accounts of the expedition.

We visited Isla Clarión from 19 May–6 June, 2013 with the specific intent of searching for *Hypsiglena*. Isla Clarión is a Pliocene seamount island 8.5 by 3.7 km, approximately 1100 km west of Manzanillo, Colima, México. We actively searched at night with headlamps between ∼1930–2400 hrs, mostly in suitable rocky habitat (but also some brush and sandy areas) on several nights prior to the full moon (24 May 2013) and resuming several nights after the full moon. During daytime hours, while monitoring other terrestrial reptiles, we opportunistically turned rocks and other suitable cover objects that were likely to contain *Hypsiglena*.

### Laboratory and Sequence Data Protocols

Extractions of genomic DNA were performed on an AutoGenprep 965 (2011 AutoGen, Inc.), using standard phenol manufacturer protocols. Genomic DNA was eluted in 100 µl of AutoGen R9 re-suspension buffer. Polymerase chain reactions were performed in 10 µl reactions for mtDNA loci using primers and PCR conditions as follows: ND4+ tRNA^His–Ser^ (here after referred to as “ND4 data”)-*HypNad4f1* and *HypLeu2r1* [15], ND5-*Leu2f1* and *nad5r1* [17], 16S-*L2510* [18] and *H3056* [19], and CO1-ReptBCF-R [20]. Cycle-sequence reactions were performed in both directions using PCR primers and BigDye Terminator v3.1 Cycle Sequencing Kit’s in 0.25×10 µl reactions and run on an Automated ABI3730 Sequencer (2011 Life Technologies). Raw chromatograms were edited in Sequencher v5.1 (2012 Gene Codes Corp.), complementary strands were aligned, and protein encoding regions were inspected for translation. Edited sequences were aligned by amino acid sequence, stem and loop alignments for ribosomal and tRNA loci followed previous studies [15]–[16]. All new samples were sequenced for the ND4 locus and new unique haplotypes were aligned and analyzed with 110 previously published *Hypsiglena* haplotypes [15]. Data for additional loci (ND5, 16S, and CO1) were collected from two of the new samples (and two previously collected samples; see Results) and were aligned to and analyzed with previously published, complete mitochondrial genome data [16], here after referred to as the “mt-genome data.” All new sequences were deposited in GenBank under the accession numbers KF548588–KF548611.

In addition to the data above, we collected two novel mt-genomes, one from an Isla Clarión sample and one from Isla Santa Catalina, using an Ion Torrent Personal Genomic Machine Sequencer (PGM). The mt-genomes were amplified via PCR in two pieces to contain duplicate control regions in separate libraries [16] using Biolabs LongAmp Taq (M03235) with the *HypNad4f1–H3056* and 16S-*L2510–HypLeu2r1* primers above. Approximately 2 µg of total PCR product was digested with RNase for 2∶30 m at 37°C, and then sheared using the QSonica Q800R Sonicator for 3 min at 30% amplitude (10 s on and 10 s off). Sheared DNA was recovered using Serapure/Sera-mag SpeedBeads. Recovered DNA quality and quantity was estimated using agarose gel and the BioTek Epoch Microplate Spectrophotometer, respectively. Approximately 80 ng of sheared total DNA was used for library preparation using the NEBNext Fast DNA Library Prep Set for Ion Torrent (Catalog Number E6270L) following the manufacturer’s protocol. Briefly, the sheared DNA was end-repaired, barcode adaptor-ligated and amplified for 10 cycles using provided primers. The amplified libraries were size-selected between 270 and 310 bp using BluePippin (1.5% Dye-free agarose cassettes with R2 internal marker) and quantified using the Ion Library Quantification Kit (Catalog Number 4468802) following manufacturer’s protocols.

We multiplexed with 8 additional libraries not pertinent to this study. Approximately 26 pM of the barcoded 14 libraries was used for template preparation on the Ion OneTouchTM 2 following the manufacturer’s instructions. Un-enriched templates were quantified on the Qubit using the Ion Sphere Quality Control Kit (Catalog Number 4468656) followed by template enrichment with Ion TouchTM ES. Sequencing was performed on the PGM using the Ion PGM 200 Sequencing Kit (Catalog Number 4474004). The combined libraries for each *Hypsiglena* yielded 235,341 (CNAR-IBH 28127) and 434,503 (MVZ 164935) sequences, with most reads between 180–240 base-pairs in length. These sequences were first trimmed and assembled into contigs in Geneious 7.0.6 (Biomatters Ltd, 2005–2013) using “De Novo Assembly, Medium Sensitivity/Fast” (other settings at default, then contigs were “Mapped to Reference” (to assemble), then aligned, and inspected for translation based on previously collected *Hypsiglena* mt-genomes [16]. The raw reads were secondarily mapped onto the complete mt-genomes and showed an average coverage >200 reads. The complete genomes were aligned with previously published mt-genomes [16], annotated, and protein coding regions were inspected for translation in Geneious and tRNA structures were verified in the alignment following previous data [16]. We found no variation in gene order within *Hypsiglena*. The two novel mt-genomes were deposited in GenBank under the accession numbers KJ486458 (CNAR-IBH 28127) and KJ486459 (MVZ 164935). The raw Ion Torrent PGM reads were submitted to NCBI SRA (SAMN02725465–66).

### Phylogenetic Analyses

Maximum likelihood phylogenetic analyses were conducted in RAxML v7.7.7 [21]. The ND4 data were partitioned by codon and tRNAs (four partitions) and the GTR+gamma substitution model was applied. A rapid bootstrap analysis (1000 pseudoreplicates) and search for the best-scoring ML tree in a single run was conducted with the rapid hill-climbing algorithm [22] and a backbone constraint tree enforced based on the topology of the previously published mt-genome data [16]. The backbone method enforces the relationships among the individuals with complete mt-genome data (based on unequivocal results from those data), while relationships among remaining individuals with ND4 data were explored. We enforced the backbone constraint as in the previous study [16] because the ND4 data alone reveal a slightly different topology compared to the complete mt-genome data (see below). We then conducted a standard bootstrap analysis in RAxML with 1000 replicates and drew the bootstrap bipartition information on the best ML tree; these values are reported. Then, the newly collected ND4, ND5, 16S, CO1, and new mt-genome data were aligned with previously published mt-genome data [16] and were analyzed with likelihood analyses identical to the ND4 data, but without any topological constraints and with different partitioning. Likelihood analyses of the mt-genome data were conducted in 39 partitions, one partition for each of the 13 protein encoding loci, two partitions for the rRNA genes (12S and 16S), one for the control region, one for origin of light strand replication, and each of the 22 tRNA genes were in their own partition. Gaps in tRNA and ribosomal genes were treated as missing data in all analyses. A 254 base-pair section of the control region was excluded from all analyses because of the ambiguous alignment, particularly with the outgroup specimens [16]. The genera *Sibon, Leptodeira,* and *Pseudoleptodeira* were used as outgroups for the ND4 analyses and *Imantodes* was also included as an outgroup for the mt-genome analyses, the same as previous studies [15]–[16]. Previous analyses of the complete mt-genome data [23] revealed that each gene, when analyzed separately, showed a slightly different topology than the topology resolved from the entire mt-genome data combined (with the exception of ND5), but differences were not strongly supported. For instance, the ND4 data, when analyzed separately, resolved the “Cochise clade” sister to a “Desert+Coast” clade with 81% bootstrap support [23], which is why we enforced the backbone constraint topology from the complete mt-genome data on the ND4 data in previous studies [16], [23] and in this study. Alignments, methods, and tree files from this study were deposited in TreeBase: http://purl.org/phylo/treebase/phylows/study/TB2:S15461.

## Results

### Sampling Results

The holotype of *H. o. unaocularus* (AMNH R62756) represents a unique form with a distinct color pattern and scalation distinguishing it from other *Hypsiglena*. It has a single post-ocular scale on either side of the head, whereas the vast majority of all other *Hypsiglena* examined have two post-ocular scales, save a few rare exceptions from throughout the range of the genus [11]. Beebe’s locality data appears to be in order, in several places his notes indicate that the *Hypsiglena* specimen was collected from Isla Clarión, at one point distinguishing it from the Clarión Racer (*Masticophis anthonyi*) as the “Small” and “Large Clarión Snake[s]” and under *Hypsiglena* indicating it as “New to Island.” Additionally, in the last chapter of his book *Zaca Venture* [10], an account of the Zaca Expedition, Beebe indicates finding a snake different from the Clarión Racer. After recounting observations of sea turtles laying eggs on the beach at night, in Sulphur Bay, Clarión Island, he states:

*“We walked on, flashing the light all around. Not far from the water on the black lava I saw a small dark brown snake. It seemed to be unlike the one I had found in daylight, having lines of black spots on the body, so I picked it up and cached it in my shirt.”* pg. 282 [10].

We arrived to Isla Clarión on 19 May 2013, with book in hand we inferred where Beebe collected the nocturnal snake based on his daytime photo of sea turtle tracks in Sulphur Bay, Fig. 23 of *Zaca Venture* [10]. That night we found two individuals of *Hypsiglena* on a small lava rock-covered knoll, near the water in Sulphur Bay. Over a 15-day period we found 11 individual nightsnakes (*Hypsiglena*) on various parts of the south-central portion of the island, all were found active at night, mostly between 2200–2300 hrs, and all but one were found in black lava rock habitat; one individual was found in a brush area with sandy substrate less than 150m from rocky habitat. Of all the specimens observed, nine individuals had one post-ocular scale on both sides of the head; two individuals had one post-ocular on one side and two on the other. All specimens had one row of large dorsal body blotches, three rows of smaller lateral blotches, and darker background coloration (in between the body blotches) different from most other *Hypsiglena* [11]. In addition, we observed that these individuals contained a unique nuchal (head and neck) color pattern, a useful character in distinguishing among species of *Hypsiglena* [11], [15]. In this population, the posterior end of the post-ocular stripe extends upward (dorsally) prior to approaching the lateral neck markings, whereas in most other *Hypsiglena*, the post-ocular stripe is either continuous with, tapers to a point, or turns downward (ventrally) prior to approaching the lateral neck markings.

### Phylogenetic Analyses

**Figure 1:**
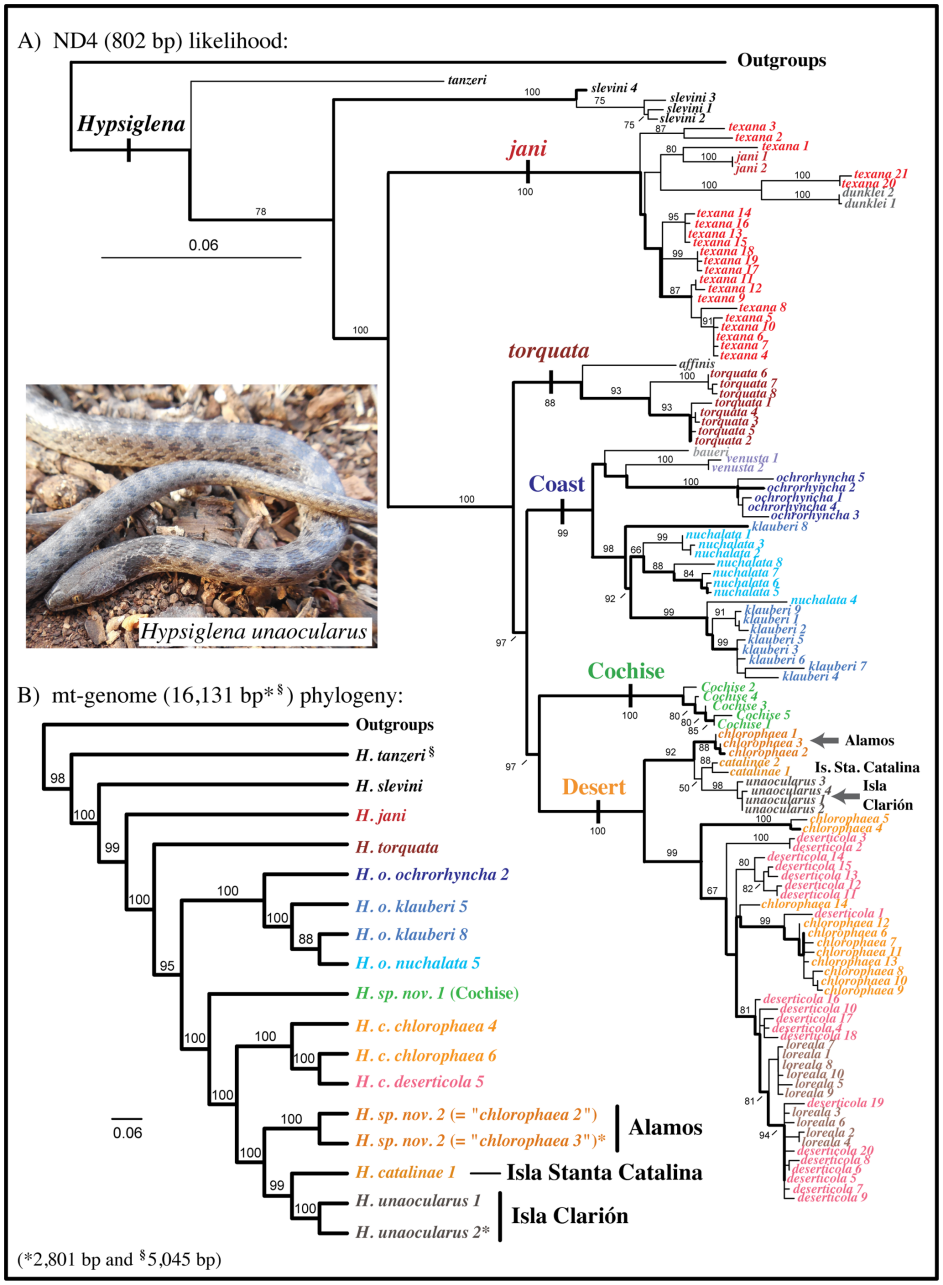
Maximum likelihood phylogenies of *Hypsiglena*, based on ND4 data (A) and mt-genome data (B). Thick lines in (A) show backbone-enforced topology (see text), haplotype names and numbers, and clade names are from Mulcahy (2008). Branch numbers represent bootstrap values. Photo of CNAR-IBH 28131 (DGM).

**Figure 2:**
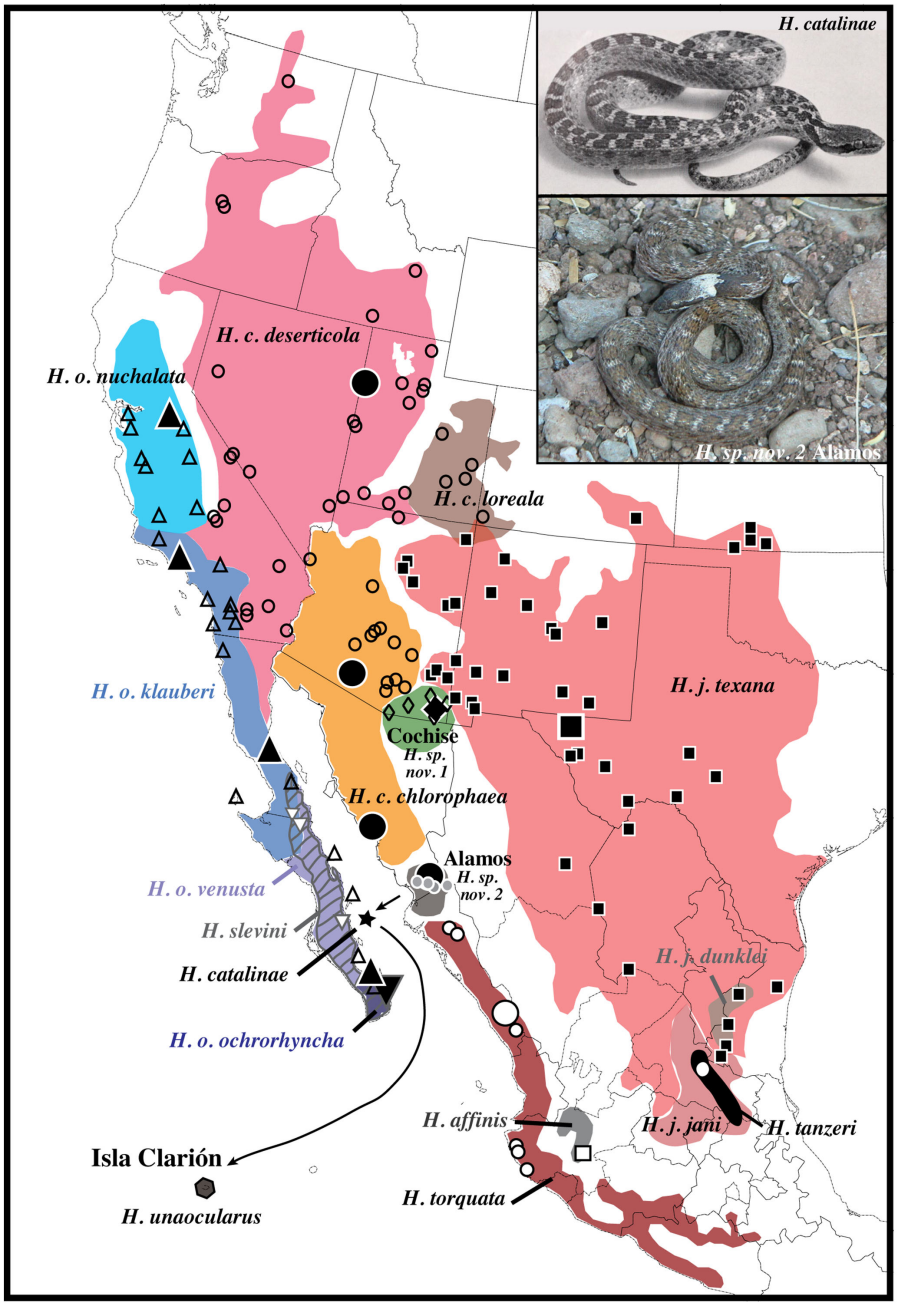
Geographic distribution of *Hypsiglena*. Colored ranges match colored clades in Figure 1 and follow prior studies, where details on previously collected samples and taxonomy can be found (Mulcahy 2008; Mulcahy and Macey 2009). Small and large symbols represent ND4 and mt-genome samples. Arrows show colonization from the Alamos area to Isla Santa Catalina (star), then to Isla Clarión (hexagon). Photos: top by D. Clites [26] with permission, bottom UTA-R 54569 (J. Meik).

Among the 11 individuals, we discovered four unique haplotypes that differed at four positions among the ND4 locus [*unaocularus* 1 (CNAR-IBH 28127, 28131; USNM Herp Image 2821); *unaocularus* 2 (CNAR-IBH 28128–30, USNM Herp Image 2816, 2818, 2820); *unaocularus* 3 (USNM Herp Image 2817); and *unaocularus* 4 (USNM Herp Image 2819)]. Maximum likelihood analyses resolved the four Clarión haplotypes in a clade sister to a clade containing haplotypes from Isla Santa Catalina, México (in the Gulf of California), with the Isla Clarión+Isla Santa Catalina clade sister to haplotypes from the Alamos area (*chlorophaea* 1–3) near the Sonora–Sinaloa state border in México (Figs. 1–2). Bootstrap support for the three populations being closely related was strong (92%), but the relationships among the three populations were weakly supported (50% Fig. 1A). Therefore, we included new sequence data for the ND5, CO1, and 16S genes (in addition to ND4) from one individual from Isla Clarión (CNAR-IBH 28128), one from the Alamos clade (BYU 42373), and CO1 for *H. tanzeri* (TCWC A-2055), and combined this with the new mt-genomes from Isla Clarión and Isla Santa Catalina and the previously published mt-genome data, which included a complete mt-genome of one individual in the Alamos clade [16]. Likelihood analyses of the mt-genome data resolved a topology identical to the ND4 data alone, but with strong support for the Isla Clarión+Isla Santa Catalina relationship (99%), with the two sister to the Alamos clade (100%; Fig. 1B).

Given the geographic distributions of these populations, the likelihood of any current gene flow is low. They are also diagnosable based on morphological characters and show fixed differences in mtDNA sequence data, suggesting they have coalesced at the morphological and genetic levels. Therefore, we recognize these populations as distinct species following the generalized lineage concept of species [24] and evolutionary species criteria [25]. *Hypsiglena unaocularus* (Tanner) [11] is restricted to Isla Clarión, México and is distinguished from other populations of *Hypsiglena* in that most individuals observed have one post-ocular scale, they have a distinct nuchal pattern where the post-ocular stripe extends dorsally before approaching the lateral neck blotches, one row of large dorsal blotches, three rows of small lateral blotches, and are dark in overall background coloration. *Hypsiglena catalinae* (Tanner) [26] is restricted to Isla Santa Catalina, México and is distinguished from other populations by having one row of large dorsal blotches, two rows of small lateral blotches, and is light in overall background coloration. The population from the Alamos area, near the Sonora–Sinaloa border in México has troubled systematists for decades because it appears morphologically intermediate between populations of *H. chlorophaea* to the North and populations of *H. torquata* to the South [11], [27]–[29], yet it is genetically distinct. Therefore, we tentatively refer to it as “*Hypsiglena sp. nov. 2″* and will provide a formal description of this species in a revision of the genus based on morphological and molecular data that is currently underway by the senior author of this study.

## Discussion

### Conservation Concerns

The rediscovery of the endemic Clarión Nightsnake highlights the importance of focused biodiversity surveys and the concern of the Islas Revillagigedo for active conservation management. Properly identifying species numbers and occurrences are first steps in conservation efforts of documenting biodiversity and quantifying extinction events [2]. Recently, several classification schemes have been proposed to the causes and significance of “extinctions” followed by rediscoveries [6], [30]. The case of the Clarión Nightsnake represents a unique scenario better described as “lost” and rediscovered [5], which falls under a combination of current classifications and several possible reasons for why it has gone undetected for so many decades. It nearly fits the “type specimen” criteria of Scheffers et al. [6], where a specimen hasn’t been seen since the type specimen was collected, which is the case for the Clarión Nightsnake. However, there are several reasons that make it different and why it has gone undetected for so long.

First, the Clarión Nightsnake was never declared extinct under any criteria [6], [30], but instead was dismissed as a locality error after not being seen for two decades [12]. Secondly, Isla Clarión is very remote and is only accessible through military escort, therefore biologists do not frequently visit the island. Further, *Hypsiglena* is not a commonly studied species (i.e. not much attention is given to this taxonomic group), and its secretive, nocturnal behavior makes it difficult to detect even when active efforts are made in general herpetofaunal surveys [9]. Therefore, the case of the missing Clarión Nightsnake involves all four of the socio-ecological factors proposed [30]. Nevertheless, its rediscovery is meaningful and noteworthy because we observed what appears to be a viable population, rather than a dwindling one, which is often the case in recent rediscoveries [5]. Finally, based on multiple lines of evidence we recognized this lineage as a distinct species, fitting the “genetic rediscovery” type of Scheffers et al. [6], such that where a subspecies was lost, a species was found. Though we revealed a potentially viable population by observing nearly a dozen individuals over a two-week period, the remoteness of this fragile island ecosystem makes it particularly susceptible to invasive species, thus warranting conservation management and protection for the Clarión Nightsnake.

The Islas Revillagigedo are small group of seamount islands that rose independently from fracture zones along the eastern edge of the Pacific Plate of North America. Often compared to the Galápagos [13], [31], their remoteness and isolation encourage evolution and speciation in plant and animal colonists. Isla Clarión is the oldest island in the group (Pliocene) and it is the furthest from the mainland, ∼1100 km west of Manzanillo, Colima, México. In 1994, the Islas Revillagigedo were designated a biosphere reserve by the Mexican government because of the large number of endemic plants and animals [32]. This archipelago is also considered an area of diversity and endemism for Mexican herpetofauna on the basis of discrepancy analysis, which compares the location of high biological richness areas with the locations of protected areas [33]. Invasive species threaten faunal components of these islands, including direct predation by feral cats on birds [32], [34]–[35] and lizards [36] on Isla Socorro. Introduced pigs, sheep, and rabbits have caused major changes in vegetation on these islands [31], [37], particularly the near obliteration of cactus on Isla Clarión [31].

Prior to this study, the terrestrial reptile fauna of the Islas Revillagigedo was thought to consist of two endemic lizards, *Urosaurus auriculatus* on Isla Socorro, *U. clarionensis*, and an endemic snake *Masticophis anthonyi* also on Isla Clarión. Our confirmation of *Hypsiglena unaocularus* on Isla Clarión identifies an additional endemic snake and truly represents a rediscovery. This is not simply the case of elevating a known inhabitant to the specific-level, as is the case for *H. catalinae* on Isla Santa Catalina. *Hypsiglena* had not been observed on Isla Clarión prior to 1936, and was essentially lost to the scientific community until now [13]–[14]. We recommend future conservation management strategies incorporate our new discovery. Efforts should be made to prevent additional introductions of invasive species. The introduction of feral cats on Isla Clarión would be devastating to the reptile community, perhaps even more so than on Isla Socorro because of the much smaller size of Isla Clarión. Although we did not observe any stomach contents or predation events by *H. unaocularus,* lizards are known to make up a large part of the diet of mainland *Hypsiglena* [38]. Therefore, *U. clarionensis* probably constitutes the major food source for *H. unaocularus,* but it is also possible that the remarkably large crickets on Isla Clarión [39] make up part of their diet as well. The introduction of feral cats could pose a double threat to *H. unaocularus*, not only by direct predation but also by affecting the main food source. Efforts should also be made to continue the eradication of current invasive species and to restore the island’s natural vegetation. Future studies should also be conducted to directly monitor *H. unaocularus.* We know very little about the abundance, distribution, and ecology of *H. unaocularus,* and its role in this fragile ecosystem.

Morphological data, geography, and phylogenetic analyses of genetic data reveal that the Clarión population represents a distinct species–*H. unaocularus* [11]. This species belongs to a clade with two other species of *Hypsiglena;* one from Isla Santa Catalina (*H. catalinae*), and a newly identified species from the Sonora–Sinaloa border in México (“*H. sp. nov. 2″*). Most importantly, we bring the presence of these species–that were otherwise unknown–to the attention of managers and conservation biologists currently monitoring vertebrates in these areas. The recognition of the Isla Santa Catalina population at the specific-level makes this *H. catalinae* another island endemic. Isla Santa Catalina contains at least eight other endemic reptile species [40]. The Rio Fuerte drainage and Alamos area near the Sonora–Sinaloa state border in México is also an area of biological interest, it represents a major transition zone between the Sonoran Desert, the Sinaloan Thornscrub, and Tropical Deciduous Forest biomes [27]–[28], [41]. Therefore, this area represents a major contact zone for many species groups [42] and contains at least one additional recently identified endemic reptile species, the Rio Fuerte Beaded Lizard *Heloderma exasperatum* [43]. The species identified in this study should be considered “endemics” to checklists of these areas and should be included in conservation management strategies to maintain biodiversity in these unique regions.

### Biogeography

The *Hypsiglena* complex has formed a geographic ring-distribution around the Gulf of California, concomitantly with the tectonic formation of the Baja California Peninsula [16] and occurs on many associated islands. Its broad insular occurrence demonstrates that nightsnakes are exceptional over-water dispersers [40]. The Alamos–Isla Santa Catalina relationship in *Hypsiglena* was previously identified and other reptiles show a similar distribution pattern [15]. Isla Santa Catalina is a deep-water, oceanic island that was never connected to Baja California; though it is closer to the peninsula than to mainland México [44]. The Rio Fuerte is a major drainage that flows into the Gulf of California from the Sierra Madre Occidental near the Alamos area [27]. Surface current patterns in the Gulf of California flow southward, whereas Pacific surface currents flow westward from the Colima–Guerrero area [45]. Direct interpretation of the phylogeny suggests that Isla Santa Catalina was colonized from the Alamos–Rio Fuerte area, and that Isla Clarión was later colonized from the Isla Santa Catalina population (Fig. 2). However, an alternative interpretation is that Isla Clarión was also colonized by flotsam expelled from the Rio Fuerte region, and the resulting topology is a result of incomplete lineage sorting of the mtDNA. A Sonora–Isla Clarión relationship is also seen in the lizards (*Urosaurus*), where the two species on the Islas Revillagigedo are more closely related to a Sonoran species than they are to more southern mainland México or Baja California species [46]. The rediscovery of the Clarión Nightsnake provides a more complete understanding for general biogeography of western North America.
